# Artificial intelligence in mental health care: a scoping review of reviews

**DOI:** 10.3389/fpsyt.2026.1688043

**Published:** 2026-03-03

**Authors:** Mohammad S. Abu-Mahfouz, Sarah AlFehaid, Hala M. Burqan, Rabie Adel El Arab

**Affiliations:** Almoosa College of Health Sciences, Al Ahsa, Saudi Arabia

**Keywords:** anxiety disorders, artificial intelligence, diagnosis, digital therapeutics, mental health, mood disorders, predictive modelling, screening

## Abstract

**Background:**

Artificial intelligence (AI) is rapidly entering mental health care, but most models remain proof-of-concept, with limited external validation and substantial risk of overfitting.

**Methods:**

This scoping review of reviews adhered to the PRISMA-ScR checklist and Joanna Briggs Institute guidance. We searched MEDLINE, Embase, PsycINFO, and IEEE Xplore. Eligible publications encompassed systematic, scoping, narrative, integrative, meta-analytic, and patent reviews. Findings were synthesised thematically.

**Results:**

Thirty-one reviews were included. Evidence concentrated on depression and anxiety; schizophrenia, bipolar disorder, perinatal mental health, autism spectrum conditions, older adults, nurses, and allied professionals were under-represented. Across screening, diagnosis/classification, and risk prediction, high accuracy was frequently reported under internal validation; in prior syntheses, typical internal AUCs clustered around ≈0.80–0.88 whereas externally or prospectively validated performance was scarce and typically attenuated. Signals were strongest for narrow, feedback-rich tasks, with greater decay for general-purpose models and longer prediction horizons. Conversational agents produced small-to-moderate short-term improvements in depressive symptoms (SMD ≈0.2–0.6); effects for anxiety and stress were smaller or inconsistent and varied with comparator stringency, follow-up (≤8–12 weeks vs longer), and the degree of human guidance. Most chatbot evaluations were short and small-scale, with few randomized or pragmatic trials and limited data on durability beyond 12 weeks. Real-world implementation was limited; several reviews identified usability and electronic health-record integration as prerequisites for adoption, and explainability alone rarely conferred actionability without clinician training. Ethical readiness was incomplete: privacy and bias were commonly discussed, but accountability, post-deployment monitoring, and crisis-escalation protocols were inconsistently specified. Economic evaluations were uncommon and rarely accounted for integration, maintenance, or re-training costs. Workforce outcomes (literacy, confidence, readiness) were infrequently measured. Internal and external metrics were not pooled.

**Conclusions:**

AI applications span the mental-health care continuum but remain early in translation. Performance that appears strong under internal validation often attenuates on external or prospective testing; symptomatic gains are concentrated in depression/anxiety and may diminish over longer follow-up; and adoption is constrained by usability, EHR integration, and incomplete governance. The cross-review signal highlights consistent gaps in accountability, post-deployment monitoring and crisis escalation, equity reporting, workforce readiness, and life-cycle economics (including integration, monitoring, and re-training). Addressing these gaps through externally validated and monitored deployments, routine content/guardrail audits for chatbots with human escalation, predefined subgroup performance and bias auditing, and implementation strategies that pair explainability with clinician training and measure workforce endpoints would better align the evidence base with safe, effective, and sustainable clinical use.

## Highlights

- External validation is imperative.
  Multicentre studies and independent test sets must become standard to confirm real-world performance and avoid overfitting performance often degrades markedly on independent cohorts.
- Broaden clinical and demographic scope.
  Future work should extend beyond mood and anxiety to severe psychiatric conditions (schizophrenia, bipolar disorder) and include under-represented groups (older adults, perinatal women, ethnic minorities, nursing/allied professionals).
- Embed implementation science.
  Phased, mixed-methods evaluations including feasibility, acceptability, hybrid effectiveness–implementation trials, and co-design with end users are essential to translate AI prototypes into routine care.
- Adopt AI-specific reporting guidelines.
  Evaluations of AI interventions must follow CONSORT-AI extension to ensure transparent, reproducible reporting of the AI component, human–AI interaction, error analyses, and bias assessments.
- Incorporate robust crisis-management protocols.
  Monitoring and telehealth tools must build in predefined escalation pathways and duty-to-warn features to safely manage users in acute distress a gap consistently noted across reviews.

## 1 Introduction

Mental health disorders are among the leading causes of disability worldwide and their prevalence continues to rise despite socio-economic development. For instance, depression and anxiety are not only debilitating but also associated with increased risk of suicide and premature mortality (1). Globally, more than one in seven (≈14 %) adolescents aged 10–19 years’ experience a mental disorder, with depression, anxiety, and behavioural disorders among the leading causes of illness and disability in this age group (2). Yet, even in high-income countries, the supply of trained mental-health professionals is insufficient; a dearth of mental-health providers in most countries remains a key barrier to delivering evidence-based care (1). In 2019, the World Health Organization estimated that one in eight people globally lived with a mental disorder and 301 million people had an anxiety disorder (3), underscoring the magnitude of unmet need.

Modern mental health care is increasingly exploring artificial intelligence (AI)–driven tools for assessment, intervention, and service delivery. However, mental health as a domain needs clear delineation from neurological or neurodevelopmental disorders to ensure conceptual clarity (4).

‘Mental health’ is a state of well-being in which individuals realize their abilities, can cope with the normal stresses of life, work productively, and contribute to their community (5). We distinguish mental health conditions (e.g., depression, anxiety, schizophrenia) from neurological and neurodevelopmental disorders, which primarily affect brain function (6). For example, autism and ADHD are neurodevelopmental disorders (7), while dementia and Parkinson’s disease are neurological disorders (8); these are not considered mental health conditions here unless they involve mental health outcomes.

In this context, AI-enabled health technologies distinct from non-adaptive digital therapeutics have been promoted as scalable tools to enhance access, personalise treatment, and augment clinician capacity. These applications draw on natural language processing, machine learning, and deep learning models to interpret speech, text, and biometric signals, deliver adaptive chat-based CBT, predict relapse, or recommend treatments (9–11). Large language models (LLMs) such as ChatGPT, have catalysed global interest in conversational agents for mental health; review shows that users value their 24-hour accessibility and non-judgemental support (12). The practitioner community also recognises the potential of AI: an international survey of 392 psychotherapists and psychiatrists found that AI technologies could help bridge the widening gap between demand and supply by facilitating early detection, personalising interventions and automating administrative tasks (13). However, clinician familiarity and adoption intentions remain low, highlighting the need for rigorous, externally validated evidence and targeted training (13).

Despite the proliferation of AI prototypes, critical concerns temper enthusiasm. Many systems are trained on datasets that underrepresent women, older adults, and minority ethnic groups. This bias can degrade performance and perpetuate inequities, even if the data appear representative (14, 15). Transparency and fairness are therefore ethical imperatives: AI tools must provide accurate diagnoses and treatments for all mental health service users regardless of their social status or ethnicity (16, 17). Moreover, the regulatory landscape is poorly defined. Current frameworks, largely adapted from software-as-a-medical-device regulations, are inadequate because AI systems evolve autonomously as they encounter new data (18, 19). A recent review called for global regulatory convergence to ensure safety, privacy, and accountability, warning that without clear oversight, liability and duty-of-care questions remain unresolved (19).

Evidence quality is another major limitation. A structured review of telepsychiatry and AI found that most publications rely on small, homogeneous samples, internal cross-validation, and lack control groups or clinical blinding, thereby limiting generalizability (20). There is a notable absence of multicentre randomized controlled trials assessing real-world effectiveness and safety of AI-based mental health interventions. Transferability to vulnerable subgroups; older adults, perinatal women, adolescents, or ethnic minorities remains largely untested, and ethics-by-design principles are seldom embedded in model development (20). These gaps underscore the risk of premature deployment of AI tools without adequate validation, transparency, or governance.

The research landscape is also fragmented. To the best of our knowledge, no synthesis has comprehensively mapped AI applications across the entire mental health care continuum (screening, diagnosis, monitoring, therapeutic intervention, and decision support) while appraising both methodological rigour and governance issues. This fragmentation hampers cross-disciplinary learning and leaves stakeholders, clinicians, patients, regulators, and developers without an integrated appraisal of benefits, limitations, and research priorities. The lack of a consolidated evidence base obscures the extent to which AI can address the mental health treatment gap and what safeguards are required to prevent harm. Recognising these limitations, we map review-type evidence across mental healthcare and explicitly separate internally derived performance from externally validated performance, account for review-level overlaps, and temper claims that rely on narrative or integrative sources without formal appraisal. Given the rapid proliferation of reviews on generative AI and the heterogeneous ways in which technical, ethical and clinical issues are addressed, we undertook a scoping review of reviews to map and synthesise higher-level evidence across these domains. Scoping reviews are recommended when the purpose is to determine the breadth and depth of available evidence, clarify definitions and concepts, and identify knowledge gaps (21, 22). Moreover, a scoping review of reviews is indicated when existing reviews are numerous but narrow in focus; synthesising them provides a holistic perspective and avoids duplication (22–24). In this review, we focus on mental health conditions in the conventional psychiatric sense – e.g. depression, anxiety, bipolar disorder, schizophrenia, post-traumatic stress disorder (PTSD), substance use disorders – explicitly excluding neurological diseases such as Alzheimer’s dementia and other neurodegenerative conditions, as well as primary neurodevelopmental disorders like autism spectrum disorder (ASD), unless these are directly tied to mental health interventions or outcomes. This scope is consistent with recent conceptual analyses distinguishing mental health problems from neurological conditions (e.g., treating ASD as an NDD distinct from mental illness). By defining our population and scope in this way, we ensure that the AI applications reviewed are relevant to mental health care (e.g., diagnosis, treatment, or monitoring of psychiatric conditions) rather than general neurology.

### 1.1 Aim

To comprehensively map and critically appraise artificial intelligence applications across the mental health care continuum, evaluating their performance and governance implications.

### 1.2 Objectives

1. To categorize AI-driven tools by core function (screening, diagnosis, monitoring, intervention, and decision support) and assess their reported diagnostic, predictive, and therapeutic performance metrics.

2. To chart the clinical domains, target populations, and deployment environments in which these AI applications have been studied.

3. To synthesize evidence on clinical benefits, ethical challenges, and regulatory frameworks, thereby identifying key research and governance gaps.

## 2 Methods

### 2.1 Study design and rationale

This study was conducted as a scoping review of reviews and followed methodological guidance from the Joanna Briggs Institute (JBI) (25), and the Preferred Reporting Items for Systematic Reviews and Meta-Analyses extension for Scoping Reviews (PRISMA‐ScR) reporting extension (26).

This design allowed us to integrate findings from diverse review types (systematic, narrative, integrative, and meta-analytic) and to explore convergences and divergences across technical, ethical, clinical and socio-cultural dimensions of generative AI in healthcare. By mapping the range of review evidence, we could clarify key concepts, examine how research has been conducted, and identify research gaps for future investigation (22, 25). Scoping reviews are designed to map the breadth of evidence on a topic and are particularly useful where concepts, populations or interventions are heterogeneous or emerging (21, 24, 27).

JBI recommends using the Population–Concept–Context (PCC) mnemonic for scoping reviews because it is less restrictive than the PICO framework and supports exploration of broad questions (28). Because this review sought to map and synthesise a heterogeneous body of literature on AI across the mental-health continuum, the research question was structured using a single evidence-synthesis mnemonic (see **Table 1**, below). We chose a thematic synthesis approach (29) to structure and interpret the findings, as it enables the identification and organization of recurring patterns and themes across diverse datasets. In keeping with scoping review methodology, we did not conduct independent risk-of-bias assessments or methodological quality appraisals of those reviews.

**Table 1:** Outlines the PCC components for this scoping review of reviews.

Component	Keywords/phrases for operational definition
Population	Mental health disorders (mood, anxiety, psychosis, bipolar, PTSD, substance-use, etc.) People at risk and stakeholders (clinicians, nurses, allied professionals, policymakers, ethicists) Exclude primary neurological disorders (e.g., dementia) and neurodevelopmental conditions unless linked to mental-health outcomes
Concept	AI/ML applied to mental health: NLP, classifiers (using clinical, neuroimaging or physiological data when aimed at mental-health outcomes), predictive models, monitoring Chatbots, wearables, sensor-based monitoring, decision support systems Virtual/augmented-reality interventions only when AI algorithms adapt content or track user state Exclude AI tools used solely for neurological assessment
Context	All healthcare and community settings (inpatient, outpatient, rehabilitation, telehealth, mobile/web platforms, research labs and educational environments); no geographic restrictions

### 2.2 Inclusion and exclusion criteria

The inclusion and exclusion criteria, summarized in **Table 2**, were designed to ensure that only reviews directly relevant to AI and mental health were included.

**Table 2:** Inclusion and exclusion criteria.

Criterion	Inclusion keywords/phrases	Exclusion keywords/phrases
Publication type	Reviews synthesising evidence on AI in mental health: systematic, scoping, narrative, integrative, umbrella, rapid and patent reviews	Primary research (RCTs, observational studies), commentaries, editorials, conference abstracts, news items, non-peer-reviewed reports
Population	Individuals with mental health conditions across the lifespan; people at risk; stakeholders in mental health care (clinicians, allied professionals, policymakers, ethicists, technologists); stress/burnout/resilience framed as mental-health outcomes	Studies focusing only on physical health; studies on neurological or neurodevelopmental conditions (e.g., dementia, ASD, ADHD) without mental-health outcomes or interventions
AI exposure	AI/ML techniques used in mental health care: screening, diagnosis, prediction, monitoring, interventions, decision support; neuroimaging or physiological-signal models only when intended for a mental-health outcome	Digital tools without AI (simple apps, telepsychiatry without AI); AI used solely for neurological or non-mental-health assessment; VR/AR interventions lacking an AI component
Outcomes	Outcomes related to function, performance, deployment setting, target population, clinical impact, ethical considerations or governance; must be linked to mental-health outcomes	Reports focusing only on algorithm development without mental-health performance or deployment outcomes
Time frame	Articles published from database inception up to 1 May 2025	Articles published after the search end date
Language	English language	Non-English languages

### 2.3 Information sources

A comprehensive search was undertaken in MEDLINE, Embase, PsycINFO and IEEE Xplore from database inception to first of May 2025. These databases were chosen to cover biomedical, psychological and engineering literature. No grey literature or preprint servers were searched to maintain peer-review integrity; this choice is justified given our review-of-reviews scope. Language was restricted to English because most eligible reviews are published in English; we note the potential for language bias.

### 2.4 Search strategy

Following the three-step process recommended by the Joanna Briggs Institute for scoping reviews, we began with an initial exploratory search to identify relevant articles and to analyse key indexing terms and text words. Building on these insights, we developed and executed comprehensive search strategies for each database on the first of May 2025. Controlled vocabularies (MeSH, Emtree and APA Thesaurus) were combined with a wide range of free-text synonyms to maximise sensitivity across both AI methodologies and mental health disorders. Boolean operators and truncation were used to merge concept clusters and capture variant spellings. All full search strategies were peer-reviewed using the PRESS checklist (30). The full, database-specific strategies are presented in Appendix 1.

### 2.5 Selection process

Duplicate records were removed. Titles and abstracts were exported to Rayyan (31). Prior to formal screening, a pilot calibration on a random sample of 50 citations was undertaken to harmonise understanding of the eligibility criteria; Cohen’s κ was calculated to quantify inter-reviewer agreement and exceeded 0.80, indicating near perfect agreement.

Titles and abstracts were screened in blind mode; any record judged potentially relevant by at least one reviewer advanced to full-text assessment.

Full-text articles were retrieved and assessed in duplicate using a standardised eligibility checklist. Discrepancies were resolved first through discussion and, if necessary, through consultation with a third reviewer. Reasons for exclusion at the full-text stage were documented.

**Figure 1:**
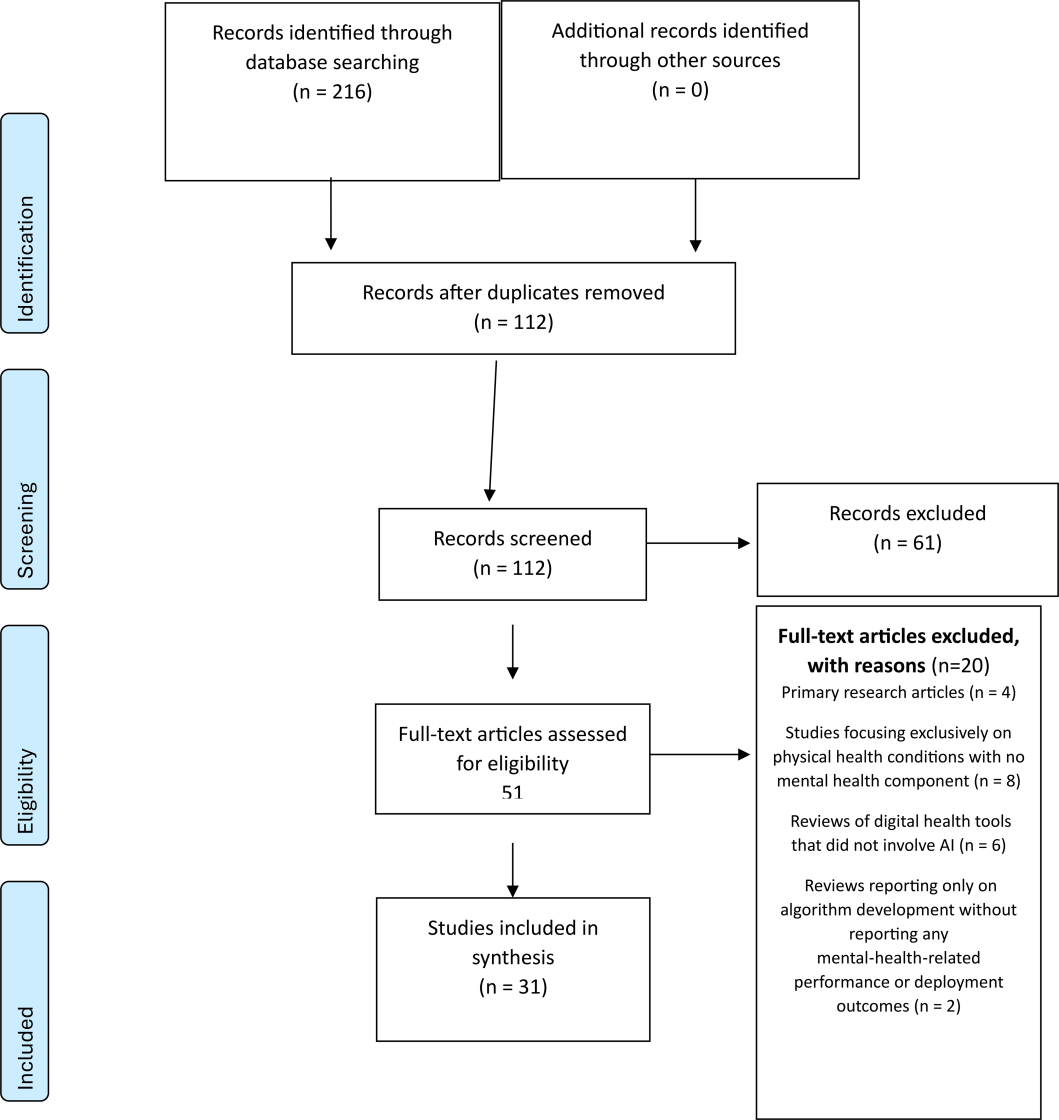
PRISMA flow diagram of study selection for the scoping review of reviews on artificial intelligence in mental health care.

A PRISMA flow diagram summarising the selection process is presented in the Results section (See **Figure 1**).

### 2.6 Data extraction

A structured data-charting form was developed in word. For each included review, we extracted the following fields: first author and year of publication; review type (e.g., systematic, scoping, narrative, integrative, meta-analytic, umbrella, or patent review); number of primary studies included; AI techniques investigated; mental health domains addressed; deployment settings; target populations; key outcome measures; ethical and governance considerations; geographic focus of the corresponding author; a concise summary of key findings; and noted limitations. Any disagreements were resolved by consensus. If needed, by third reviewer. Consistent with JBI/PRISMA-ScR guidance, we did not conduct a formal risk-of-bias appraisal of reviews; however, we extracted four review-level credibility indicators (protocol/registration, comprehensive search, duplicate screening, and use of a primary-study bias tool) to contextualize certainty.

To avoid inflated impressions of robustness, we separated all quantitative statements by review type (systematic, scoping, narrative/integrative) and validation regime (internal cross-validation vs external independent/prospective/RCT/real-world). Internal and external metrics were never pooled. We report a conservative read based on external/prospective evidence when available and flag small-study effects, short follow-up, and risk-of-bias alongside each summary. ‘Hero’ internal metrics are presented only in an internal-benchmarks sidebar, not in the abstract or headline findings. We explicitly distinguished internal (resampling/same-site split) from external (temporal, geographic, or multi-site) validation.

### 2.7 Data analysis

Data Analysis subsection to clarify how themes were derived. We performed a thematic synthesis of the data from included reviews (29, 32, 33). This process was primarily inductive, meaning we did not impose *a priori* coding frame but rather derived themes from the reported results of the reviews included. Two independent researchers (RA, MA) coded descriptive findings from each review and then discussed and merged codes into higher-order themes. Through iterative refinement, we identified five overarching domains of AI systems in mental health care. These five domains emerged from the data itself, but we note they broadly align with frameworks in the literature that classify AI applications by function (e.g., assessment/diagnosis, monitoring, intervention, etc.). If an included review’s findings touched on a predetermined category (for example, clinical decision support), we incorporated that under the corresponding theme, but we remained open to new themes (ensuring a primarily inductive approach). The final five domains thus represent a synthesis of recurring patterns across the reviews. For transparency, the team reached consensus on these domains, and any discrepancies in theme naming were resolved through discussion. If a previously published schema was relevant, we used it only after themes emerged to confirm our grouping (i.e., a mixed inductive-deductive approach), rather than to dictate it. We cite relevant literature when referring to established categories in our analysis.

## 3 Results

**Figure 1**. PRISMA flow diagram of study selection for the scoping review of reviews on artificial intelligence in mental health care.

### 3.1 Characteristics and methodological quality

Thirty-one review articles (2019–May 2025) met inclusion criteria (9–11, 34–62). Eleven PRISMA-guided systematic reviews (10, 35, 36, 38, 43, 47, 52–55, 62), including two with meta-analyses (36, 54), five PRISMA-ScR scoping reviews (39, 40, 44, 51, 56), two integrative review (41, 42), one patent-analytics review (57), and thirteen narrative, conceptual, or ethical overviews (9, 11, 34, 37, 45, 46, 48–50, 59–61). The reviews included in this review varied substantially in quality: systematic and scoping reviews used established risk-of-bias tools such as (Cochrane RoB 2, ROBINS-I, MMAT, CASP, JBI or AMSTAR 2), but only a minority reported protocol registration, duplicate screening, or comprehensive search strategies. Narrative and conceptual reviews rarely conducted any risk-of-bias assessment. Reporting of credibility indicators was uneven across reviews. This heterogeneity in methodological rigor underscores the need to interpret aggregated findings with caution. In our synthesis we treated those neurodevelopmental/neurocognitive conditions as out of scope for mental health, focusing only on any mental health aspects reported. For example, one narrative review (Sun et al., 2023) (59) listed “Alzheimer’s disease” among its target conditions, but we did not consider dementia-related findings in our analysis, as Alzheimer’s is a neurological disorder rather than a mental health condition. Similarly, an integrative review by Auf et al. (2025) (40)included autism spectrum disorder in its scope; we included this source because it also covered depression and other mental health problems, but we excluded any purely autism-focused conclusions when synthesizing mental health themes. After applying these filters, the domains and examples discussed below pertain strictly to mental health interventions or outcomes.

### 3.2 Thematic synthesis

Our thematic synthesis of thirty-one reviews yielded five overarching domains: (1) Care Continuum Functions; (2) Clinical Domains; (3) Target Populations & Stakeholders; (4) Deployment Settings; and (5) Outcomes, Benefits & Ethical Considerations. For each domain, performance metrics and effect sizes are presented descriptively without implying conclusive evidence, consistent with scoping review methodology.

#### 3.2.1 Care-continuum functions (“what AI does”)

AI in mental health care has progressed from early rule-based expert systems to a broad suite of data-driven tools spanning risk detection through therapeutic support (9, 10, 48, 49, 54, 55, 59, 60).

Screening and early detection relied predominantly on natural-language processing to analyse clinical notes, social-media posts and spoken language, while computer-vision algorithms interpreted facial micro-expressions and affective states (9, 34, 44, 47, 48, 50, 62).

Diagnostic and classification models combined neuroimaging, electrophysiological signals and linguistic features within supervised machine-learning or deep-learning architectures to differentiate major depressive disorder, bipolar disorder and schizophrenia (11, 47–49, 54, 59, 60).

Predictive modelling integrated multi-modal data; genomic profiles, electronic health records, wearable metrics and lifestyle variables using ensemble or hybrid approaches to forecast disease onset, relapse or treatment response; however, most models were trained on small, homogeneous cohorts and validated internally rather than on independent samples, leading to potential overestimation of performance (9, 11, 42, 47, 48, 54, 60).

Monitoring and telehealth augmentation leveraged wearables, ambient sensors and smartphones for real-time tracking of physiological and behavioural signals, and conversational agents-maintained engagement in mobile-health platforms (47, 48, 50, 51, 62).

Therapeutic interventions encompassed CBT based chatbots (e.g., Woebot, Wysa, Youper), guided mindfulness exercises, psychoeducation modules, virtual-reality exposure therapy and early neurofeedback prototypes (41, 43, 44, 50, 51, 55, 61).

Clinical Decision-Support Systems (CDSS) integrated into Electronic Health Records (EHRs) offered remission probabilities and treatment recommendations, but only a handful of evaluations assessed their real-world impact (40, 42, 47, 54).

#### 3.2.2 Clinical domains (“where it’s applied”)

AI applications span a wide range of psychiatric conditions, with a clear concentration on mood disorders (36, 47–50, 54, 59, 60).

Applications addressing stress and resilience were included only when explicitly linked to mental-health outcomes (for example, preventing or monitoring anxiety and depressive disorders); these studies commonly employed physiological sensors and decoded neurofeedback to detect stress responses and build coping strategies (48, 61).

Trauma and Post Traumatic Stress Disorder (PTSD) appear intermittently, often via app-based tools such as PTSD Coach or neuroimaging classifiers, although real-world implementation remains thin (40, 44, 47, 50, 55). Psychosis-related models often report accuracies approaching or exceeding 85% only in small, proof-of-concept cohorts with internal cross-validation and without independent external validation, limiting their generalizability (11, 47, 48, 54).

Pear Therapeutics’ reSET is an U.S. Food and Drug Administration (FDA)-cleared prescription digital therapeutic delivering a 12-week CBT and contingency management program via a mobile app, with Randomized Controlled Trial (RCT) evidence showing a 50% reduction in substance cravings (OR 0.48; 95% CI 0.32–0.73) (9, 47, 60).

Cognitive decline and dementia feature sporadically: Bard’s true-positive rate for Alzheimer’s identification was 89% in a zero-shot, vignette-based classification task, but it also frequently misidentified cognitively normal cases as Alzheimer’s, demonstrating low specificity (38).

Emerging niches include AI-enhanced life-crafting for student resilience, perinatal mental-health chatbots, refugee and migrant decision-support systems, and nursing-focused syntheses each represented by only a handful of studies alongside scattered work in autism, attention-deficit/hyperactivity disorder (ADHD), and eating-disorder prevention (41, 42, 44, 45, 47, 50, 51, 61).

Reviews diverged on conversational agents for anxiety and stress: some reported small-to-moderate short-term benefits (e.g., SMD ≈0.2–0.5), whereas others found attenuated or null effects after adjusting for risk of bias and engagement decay (43, 50, 51, 55). Differences in comparator stringency, follow-up (≤8–12 weeks vs longer), and guidance intensity likely explain these discrepancies (43, 51, 55, 62).

#### 3.2.3 Target populations and stakeholders (“for whom”)

AI in mental health engages a range of end users, but the inclusion beyond patients and clinicians remains uneven.

Most implementations target adult mental health service users clinical or subclinical who engage with chatbots, digital therapeutics, or monitoring apps; older adults and perinatal women are represented more sporadically (10, 36, 43, 50, 51, 54, 61).

Clinicians, including psychiatrists, psychologists, and primary-care physicians, use AI for diagnosis, risk stratification, and workflow support, while mental-health nurses and allied professionals remain under-represented in development and evaluation (40–42, 56).

Academic and Educational Populations feature in AI-enhanced life-crafting programmes that integrate goal setting with conversational agents (40, 42, 45, 54). Finally, ethicists, policy-makers, and multidisciplinary scholars shape the discourse on privacy, bias, accountability, and governance for conversational and generative platforms (35, 37, 39, 44, 53).

#### 3.2.4 Deployment settings (“where it’s deployed”)

AI tools are predominantly deployed in digital environments, with varying degrees of integration into traditional care settings:

Digital Platforms: Mobile and web applications host chatbots, symptom trackers, and resilience modules, enabling scalable, remote access (44, 50, 51, 62).

Clinical Environments: EHR-embedded CDSS and imaging suites incorporate AI models for diagnostic and treatment planning tasks (40, 42, 47, 54, 60).

Community and psychiatric rehabilitation settings remain comparatively nascent, although a few CDSS and rehabilitation models are reported (40–42, 56).

Academic & research labs: Prototype virtual reality (VR) and augmented reality (AR) interventions were included only when they incorporated AI algorithms (e.g., adaptive personalisation or real-time user-state tracking), and decoded neurofeedback trials typically occur under controlled lab conditions (41, 44, 50, 61).

Teletherapy and Hybrid Models: Across teletherapy and other digital/hybrid deployments, standardized AI–clinician workflows and clear crisis-escalation handoff protocols remain largely absent, as shown by real-world implementation evidence gaps (40) and ethical analyses emphasizing safety, responsibility, and duty-to-warn mechanisms (39).

#### 3.2.5 Outcomes of interest, reported benefits and ethical considerations

##### 3.2.5.1 Clinical benefits

Diagnostic and Predictive Performance: These values predominantly arose from internal validation; where external testing was reported, performance was lower and more variable. Graham et al. (2019) (11) report AUCs up to 0.98 and accuracies reach 97% in small, proof-of-concept cohorts, but pooled metrics reflect internal validation only; performance often degrades on independent data. In a broader synthesis, Rony et al. (2025) (54) model performance was predominantly from internal validation (typical AUC ≈0.80–0.88); externally validated performance was sparse and, where available, lower.

Symptom Reduction: A meta-analysis of AI-driven conversational agents found a moderate-to-large reduction in depressive symptoms (Hedges’ g = 0.61) but only small or non-significant effects on anxiety, stress, and overall, well-being, indicating heterogeneous benefits across outcomes (36). Farzan et al. (2025) (43) confirm similar moderate-to-large effects (g≈ 0.46–0.64) across Woebot, Wysa, and Youper trials.

Engagement and Therapeutic Alliance: Conversational agent users average Working Alliance Inventory–Short Revised scores around 3.36/5 overall and 3.80/5 on the bond subscale, closely matching values seen in human-delivered CBT (43). These patterns mirror the alliance–outcome relationship reported in human-delivered CBT, though empathy gaps remain a concern.

Workflow Efficiency: Ambient listening AI assistants have been reported in early, mainly non-psychiatric pilots to reduce documentation time and improve perceived efficiency, but mental-health-specific trials are lacking (37, 59). Economic evaluations were uncommon and, when present, typically omitted integration, interface redesign, data-protection engineering, post-deployment monitoring, and model re-training costs, limiting interpretability of reported value.

Resource optimization in nursing has largely been proposed or explored at early-stage/prototype levels (e.g., predictive relapse or staffing models), with limited evidence of routine implementation (40–42, 56). Workforce endpoints including AI literacy, confidence, readiness, and infrastructure adequacy were rarely prespecified or measured alongside clinical outcomes, constraining inference about implementation feasibility and safety.

##### 3.2.5.2 Ethical and practical concerns

Data Privacy & Security: Digital therapeutics and sensor-based monitoring carry ongoing risks of data breaches and unclear governance. Olawade et al. (2024) (9) highlight the nascent regulatory frameworks for weadata, and mobile-health data, and Meadi et al. (2025) (39) identify privacy/confidentiality as the most frequently discussed ethical theme (61 % of articles).

Algorithmic Bias & Equity: Multiple reviews note Western-centric, demographically narrow datasets and the absence of systematic fairness audits; consequently, models may underperform for marginalized populations (9, 35, 37, 39, 40, 44, 48, 54, 59).

Ethical readiness was incompletely addressed; most reviews mentioned privacy and bias, fewer specified accountability or post-deployment monitoring (35, 37, 39, 40, 62).

Explainability and Trust: Joyce et al. (2023) (53) systematically reviewed 25 studies on XAI in mental health, finding predominant reliance on post-hoc feature-importance methods such as SHapley Additive exPlanations (SHAP), Local Interpretable Model-Agnostic Explanations (LIME) and limited human-centred evaluation. Baydili et al. (2025) (48) document uneven integration of interpretable pipelines in practice.

Therapeutic Alliance and Depersonalization: Although chatbots can establish strong working alliances, Vaidyam et al. (2019) (55) caution about empathy gaps, and Meadi et al. (2025) (39) recommend blended human–AI care models to preserve therapeutic relationships, rather than unequivocally ‘advocating’ a single solution.

Regulatory & Liability Gaps: Many AI systems especially wearables and generative platforms operate in regulatory “silence.” Olawade et al. (2024) (9) describe the absence of clear oversight pathways for such tools, and Blease & Rodman (2025) (37) highlight unclear duty-to-warn protocols for generative AI in clinical contexts, underscoring the need for defined software-as-medical-device pathways and governance standards. These ethical concerns highlight the imperative for transparent, stakeholder-informed governance structures.

## 4 Discussion

Our scoping review of 31 review-type publications provides a descriptive mapping of AI across mental health care rather than definitive evidence of effectiveness. It reveals that AI is rapidly permeating every stage of mental health care: from early screening and diagnosis through continuous monitoring, intervention, and decision support. Proof-of-concept studies consistently demonstrate high diagnostic accuracy across conditions such as depression and schizophrenia, while meta-analytic syntheses report substantive therapeutic benefits in controlled trials. These advances span natural language processing models that flag distress in text and speech, neuroimaging classifiers that differentiate psychiatric diagnoses, and wearable-based systems for continuous symptom and risk monitoring. AI-powered interventions such as chatbot-delivered CBT and VR exposure therapies offer scalable adjuncts to traditional care. Despite these promising developments and AI’s potential to extend access in resource-limited settings, real-world implementation remains uneven and predominantly preliminary. While formal review appraisal is outside the remit of scoping reviews, reporting credibility indicators improves interpretability without overweighting low-rigour summaries. Synthesis across paradigms should be read as directional rather than pooled.

### 4.1 Uneven adoption across clinical domains

Consistent with major reviews, AI in mental health has overwhelmingly targeted affective disorders chiefly depression and anxiety while other areas remain at proof-of-concept stages. Farzan et al.’s (2025) systematic review of 10 CBT‐chatbot studies confirms that most evaluate depressive and anxiety outcomes (only two are RCTs), with only isolated trials addressing substance misuse or perinatal mood (43). By contrast, Sun et al. (2023) reports that AI applications in schizophrenia, bipolar disorder, PTSD, and ASD remain confined to small cohorts and prototype settings with limited external validation (59). These imbalances driven in part by the ease of standardizing depression/anxiety measures versus the complexity of serious mental illnesses underscore the need for multicentre RCTs, broader stakeholder engagement (including nursing and rehabilitation), and regulatory harmonization to extend AI’s reach across the full psychiatric spectrum. These patterns highlight the urgent need for multicentre RCTs and broader stakeholder engagement, including nursing and rehabilitation professionals to extend AI’s reach across the full psychiatric spectrum.

### 4.2 Methodological and validation challenges

A pervasive limitation across AI in mental health is the reliance on narrow, single-site datasets with only internal validation. Many machine-learning classifiers boast AUCs or accuracies above 85–90% in development cohorts, for example, Baydili et al. 2025 (48) report an electroencephalography (EEG)-based convolutional neural network (CNN) achieving an in-sample AUC of 0.99 that declined to 0.73 on an external data, yet performance often deteriorates markedly on independent test sets. Small sample sizes and the absence of multicentre or external trials artificially inflate best-case metrics and hinder reproducibility. Such performance decrements indicate overfitting and underscore the need for multicentre external validation. Moreover, only a minority of reviews and the primary studies synthesize adhere fully to methodological safeguards such as protocol registration, duplicate screening, comprehensive bias assessment, and open data/code sharing. Without standardized reporting frameworks such as Consolidated Standards of Reporting Trials for Artificial Intelligence (CONSORT-AI), whose adoption remains limited despite clear recommendations (39, 46), reliable meta-analysis and cross-study comparison remain out of reach.

### 4.3 Equity, bias, and generalizability

To ensure equitable performance, future research must prioritize dataset diversity and embed systematic bias-detection and mitigation measures such as subgroup performance benchmarking, synthetic data augmentation, and transparency in model decision pathways throughout model development.

Most AI studies in mental health rely on *post hoc* feature-importance techniques (e.g., SHAP, LIME) for explainability yet rarely assess whether these visualizations actually improve clinician understanding or trust. In psychiatry and mental health settings where nuanced clinical judgment and the therapeutic alliance are paramount such “black-box” models impede uptake, as ethical reviews emphasize the need for transparency and accountability.

### 4.4 User experience and acceptability

Both patients and providers generally report favourable experiences with AI tools: chatbots and apps are viewed as accessible, nonjudgmental, and capable of fostering a therapeutic alliance akin to human-led interventions (43, 55). Users value real-time feedback and 24/7 availability features that enhance engagement, particularly for those facing barriers to in-person care (34, 43, 49). However, chatbots may struggle with complex emotional nuance, raising concerns about their suitability for individuals in severe distress (39). These findings underscore AI’s role as an adjunct not a replacement for human clinicians. These summaries plausibly reflect heterogeneity in trial design and dose/engagement, rather than a single underlying effect.

### 4.5 Ethical, privacy, and regulatory considerations

Privacy and data security are paramount given the highly sensitive nature of mood logs, voice recordings, and physiological signals captured by AI-driven tools (35, 39). Yet informed consent processes and data-protection standards remain inconsistent, leaving many mental-health apps stranded in a regulatory gray area between consumer wellness products and regulated medical devices (37).

Crisis management features are underdeveloped: few systems incorporate predefined “duty to warn” protocols or escalation pathways to engage human responders (39). At the same time, questions of algorithmic liability and clinician accountability persist, with no clear legal or professional guidelines for who bears responsibility when AI outputs cause harm.

### 4.6 Recommendations and implications

Translation will not be accelerated by broader exhortations but by enforceable standards tied to measurable outcomes. We therefore propose a minimum, testable quality bar across six fronts validation, safety, equity, economic value, adoption, and accountability each linked to evidence and to concrete metrics your trials and services can report.

First, validation must be external, prospective, and lifecycle-aware. Models intended for screening, triage, or relapse prediction should demonstrate stability on temporally and geographically distinct cohorts before any live use, and post-deployment drift monitoring with pre-specified re-training triggers should be routine. Economic evaluation should mirror that lifecycle: budgets must include integration, interface redesign, privacy engineering, monitoring, and re-training, and use dynamic models that penalise performance decay rather than static, one-off projections (63).

Second, safety for conversational agents requires routine content and guardrail audits, coupled with real-time sentiment monitoring to identify distress and an auditable human-escalation pathway. These practices are supported by adjacent public-health deployments showing that regularly audited chatbots with real-time analytics can surface emerging risks (64); in mental health, they should be treated as necessary safeguards to prevent harm, then evaluated prospectively.

Third, equity must be demonstrated rather than declared. Every evaluation should publish subgroup performance (at minimum by sex, age band, ethnicity where lawful, language, and comorbidity), run scheduled bias audits, and use federated or privacy-preserving data-sharing where centralisation is infeasible, alongside capacity-building in under-represented settings to avoid systematic performance gaps (64).

Fourth, adoption is a socio-technical outcome. Evidence from infection-control AI shows that usability and EHR integration are adoption gatekeepers, and that explainability (for example, SHAP) improves actionability only when accompanied by targeted clinician training and embedded workflows; mental-health deployments should therefore budget for interface co-design, training, and integration as first-order requirements, then measure whether these investments change behaviour (65).

Fifth, workforce readiness should be a co-primary endpoint. Systematic evidence across nursing and allied professionals shows guarded optimism but moderate literacy/readiness and infrastructure barriers; studies should therefore co-measure clinical outcomes with workforce literacy, confidence, and use, and report whether training closes the preparedness–impact gap (66, 67).

Sixth, accountability must be observable. Trials and services should document who reviews model outputs, who can override them, what happens when guardrails fire, and how patients can contest decisions; these governance elements should be reported as consistently as accuracy or symptom change (64).

To operationalise these recommendations, we specify auditable minimum standards for validation, safety, equity, economic value, adoption, and workforce readiness; the corresponding requirements and measures are summarised in **Table 3**.

**Table 3:** Deployment-readiness checklist for mental-health AI-required practices, core measures, and evidence basis.

Domain	Required practice	Core measures to report
Validation & monitoring	External, prospective evaluation; drift surveillance with retraining triggers pre-specified	Temporal & geographic external AUC/Brier change; calibration shift; time-to-retraining; safety events pre/post retraining
Economic value	Dynamic, lifecycle costings including integration, maintenance, monitoring, and re-training	ICER/QALY with scenario analyses; budget impact over 1–3 years; sensitivity to decay in performance
Safety for chatbots	Quarterly content/guardrail audits; real-time sentiment flags with human escalation	Audit non-conformances per 1,000 interactions; time-to-human contact after red-flag; adverse event rate
Equity	Subgroup performance + routine bias audits; federated or privacy-preserving data-sharing; capacity-building	ΔAUROC/Calib by subgroup; fairness metrics over time; number of partner sites added in under-represented regions.
Adoption & actionability	EHR integration; usability testing; XAI paired with training	Task completion time; acceptance/override rates; pre/post training change in correct actions based on explanations.
Workforce endpoints	Co-primary readiness and literacy outcomes with clinical endpoints	Validated literacy/readiness scales; confidence to use; sustained use; association with safety and symptom change.

The section moves beyond platitudes to a pragmatic contract: if mental-health AI is to claim clinical value, it must document durability on external data, safety under guardrails with human oversight, distributional fairness, lifecycle affordability, real clinician uptake, and a prepared workforce each evidenced with the same discipline we apply to effect sizes.

### 4.7 Strengths and limitations

This scoping review’s strengths lie in its comprehensive search strategy across MEDLINE, Embase, PsycINFO and IEEE Xplore, each strategy peer reviewed against the PRESS checklist, to capture a broad spectrum of biomedical, psychological, engineering and patent literature. Our dual-screening approach, anchored by a pilot calibration exercise that yielded a Cohen’s κ > 0.80, enhanced consistency and minimized selection bias during study identification. Moreover, by employing a thematic synthesis framework, we were able to map AI applications seamlessly across the entire mental-health continuum from early screening and diagnostic classification through continuous monitoring, digital interventions and decision support offering an integrated, panoramic perspective rarely accomplished in prior reviews.

However, several limitations temper our findings. Although many of the included reviews themselves applied established risk-of-bias tools (for example, Cochrane RoB 2, ROBINS-I, AMSTAR 2), we did not undertake our own independent methodological or bias assessments, which restricts our capacity to weight conclusions by review quality. The heterogeneity of review types (systematic, narrative, integrative, patent) and the wide variance in reported outcome measures precluded any form of quantitative synthesis or meta-analysis. Limiting our review to English-language sources introduces language and publication bias.

## 5 Conclusion

This scoping review encompasses systematic, scoping, narrative, integrative, and patent analyses reveals that AI applications have rapidly extended across the mental health care continuum. Yet the evidence base remains in early stages: proof-of-concept studies and meta-syntheses show promise but rely on small, homogeneous cohorts and lack robust external validation. Domain coverage is uneven, with mood and anxiety disorders dominating, while severe presentations and underserved populations are largely overlooked. To advance AI as a safe, equitable adjunct to clinical practice, research must adopt standardized reporting frameworks, conduct multicentre validation, embed implementation science and stakeholder co-design, and enforce proactive bias mitigation, clinician-centric explainability, and ethical and regulatory oversight.

## Data Availability

The original contributions presented in the study are included in the article/**Supplementary Material**. Further inquiries can be directed to the corresponding author.

## Glossary

ADHD: Attention−Deficit/Hyperactivity Disorder
AI: Artificial Intelligence
AMSTAR 2: A Measurement Tool to Assess Systematic Reviews 2
ASD: Autism Spectrum Disorder(s)
CASP: Critical Appraisal Skills Programme
CAMI: Community Attitudes toward the Mentally Ill (scale)
CBT: Cognitive Behavioural Therapy
CDSS: Clinical Decision Support System(s)
CNN: Convolutional Neural Network
Cochrane RoB 2: Cochrane Risk of Bias 2 tool
CONSORT−AI: Consolidated Standards of Reporting Trials for Artificial Intelligence
EEG: Electroencephalography
EHR(s): Electronic Health Record(s)
FDA: U.S. Food and Drug Administration
fMRI: Functional Magnetic Resonance Imaging
JBI: Joanna Briggs Institute
LIME: Local Interpretable Model−Agnostic Explanations
LL M: Large Language Model
ML: Machine Learning
MMAT: Mixed Methods Appraisal Tool
MRI: Magnetic Resonance Imaging
NLP: Natural Language Processing
OR: Odds Ratio
PRISMA: Preferred Reporting Items for Systematic Reviews and Meta−Analyses
PRISMA−ScR: PRISMA extension for Scoping Reviews
PTSD: Post−Traumatic Stress Disorder
QALY: Quality−Adjusted Life Year
RCT: Randomized Controlled Trial
RE−AIM: Reach, Effectiveness, Adoption, Implementation, Maintenance
ROBINS−I: Risk Of Bias In Non−randomized Studies –
RoB: Risk of Bias
SHAP: SHapley Additive exPlanations
SPIRIT−AI: Standard Protocol Items: Recommendations for Interventional Trials –
VR: Virtual Reality
XAI: Explainable Artificial Intelligence
AUC: Area Under the Receiver-Operating-Characteristic Curve
AUROC: Area Under the Receiver-Operating-Characteristic Curve
CFIR: Consolidated Framework for Implementation Research
DecNef: Decoded Neurofeedback
ED: Emergency Department
HTA: Health Technology Assessment
ICER: Incremental Cost-Effectiveness Ratio
LLM: Large Language Model.
