# Supplementary material for: Artificial intelligence in mental health care: a scoping review of reviews
Source: Front Psychiatry. 2026 Mar 3;17:1688043. doi: 10.3389/fpsyt.2026.1688043 (PMC12993279; doi:10.3389/fpsyt.2026.1688043)
Supplement: Supplementary file 1 [file Table1.docx]

**Appendix 1. presents the key terms and structure used for each database.**

| Database | Key controlled vocabulary | Free‑text search terms |
| --- | --- | --- |
| MEDLINE (Ovid) | MeSH terms: Artificial Intelligence, Machine Learning, Natural Language Processing, Mental Health, Mental Disorders, Psychiatry, Review Literature as Topic. | (“artificial intelligence” OR “machine learning” OR “deep learning” OR “natural language processing” OR “neural network*” OR chatbot* OR “conversational agent*” *OR “large language model*” OR “ChatGPT” OR “generative AI” OR “virtual reality” OR “predictive model*”) AND (“mental health” OR “mental disorder*” OR psychiatr* OR psycholog* OR depressi* OR anxiety OR “bipolar disorder” OR schizophrenia OR PTSD OR autism OR ADHD OR “substance use”) AND (review OR “systematic review” OR “scoping review” OR “narrative review” OR “integrative review” OR “meta‑analysis” OR “umbrella review” OR “patent review”). |
| Embase (Ovid) | Emtree terms: artificial intelligence, machine learning, natural language processing, neuroimaging, psychiatry, mental disease, review. | ((“artificial intelligence” OR “machine learning” OR “deep learning” OR “natural language processing” OR chatbot* OR “conversational agent*” OR “virtual reality” OR “wearable” OR “predictive model*”) AND (“mental health” OR “mental disorder*” OR psychiatr* OR psycholog* OR depressi* OR anxiety OR schizophrenia OR “bipolar disorder” OR PTSD OR autism OR ADHD OR “substance use”)) AND (review:ti,ab OR “systematic review” OR “scoping review” OR “narrative review” OR “integrative review” OR “meta‑analysis” OR “umbrella review” OR “patent review”). |
| PsycINFO (EBSCO) | APA Thesaurus terms: Artificial Intelligence, Machine Learning, Natural Language Processing, Mental Health, Mental Disorders, Psychiatry, Review. | ((“artificial intelligence” OR “machine learning” OR “deep learning” OR “neural network*” OR chatbot* OR “conversational agent*” OR “virtual reality” OR “augmented reality” OR “predictive model*”) AND (“mental health” OR “mental disorder*” OR psychiatr* OR psycholog* OR depressi* OR anxiety OR schizophrenia OR “bipolar disorder” OR PTSD OR autism OR ADHD OR “substance use”)) AND (review* OR “systematic review*” OR “scoping review*” OR “narrative review*” OR “integrative review*” OR “meta‑analysis” OR “umbrella review” OR “patent review”). |
| IEEE Xplore | Subject filters: Artificial Intelligence (controlled vocabulary); Mental Health; Review; content type = journals and conference proceedings. | (“artificial intelligence” OR “machine learning” OR “deep learning” OR “natural language processing” OR chatbot* OR “conversational agent*” OR “neural network*” OR “virtual reality”) AND (“mental health” OR “mental disorder*” OR psychiatr* OR psycholog* OR depressi* OR anxiety OR schizophrenia OR PTSD OR autism OR ADHD) AND (review OR survey OR overview). |
